# Micrognathia with temporomandibular joint ankylosis and obstructive sleep apnea treated with mandibular distraction osteogenesis using skeletal anchorage: a case report

**DOI:** 10.1186/s13005-017-0150-4

**Published:** 2017-11-10

**Authors:** Hiroshi Tomonari, Hiroko Takada, Tomofumi Hamada, Sangho Kwon, Tsuyoshi Sugiura, Shouichi Miyawaki

**Affiliations:** 1 Department of Orthodontics, Kagoshima University Graduate School of Medical and Dental Sciences, Kagoshima, Japan; 2 Department of Oral surgery, Kagoshima University Graduate School of Medical and Dental Sciences, Kagoshima, Japan

**Keywords:** Mandibular distraction osteogenesis, Micrognathia, Obstructive sleep apnea, Skeletal anchorage, Sliding genioplasty, Temporomandibular joint ankylosis

## Abstract

**Background:**

We describe the case of a 16-year-old female patient with micrognathia, temporomandibular joint (TMJ) ankylosis, and obstructive sleep apnea, who was treated with mandibular distraction osteogenesis (DO) combined with sliding genioplasty, using skeletal anchorage.

**Case presentation:**

We first performed interpositional arthroplasty, in which an interposition of fascia temporalis and surrounding fat tissue was inserted into the defect after bilateral condylectomy, increasing the maximum mouth opening from 5.0 to 32.0 mm. Subsequently, orthodontic treatment and advancement of the mandible were carried out by mandibular DO, using miniscrews and miniplates. Finally, sliding genioplasty was performed to bring the tip of the mandible forward. The total amount of mandibular advancement at the menton was 16.0 mm. An improved facial appearance and good occlusion were eventually achieved, and the apnea-hypopnea index decreased from 37.1 to 8.7. There was no obvious bone resorption or pain in the temporomandibular region, limited mouth opening (maximum mouth opening: 33.0 mm), myofascial pain or headache, downward rotation of the mandible, or lateral shift of mandibular position evident at 5 years and 6 months after mandibular DO.

**Conclusion:**

Mandibular DO using skeletal anchorage with intermaxillary elastics is useful for preventing extrusion of the upper and lower anterior teeth, thereby preventing rotation of the mandible. In addition, mandibular DO combined with sliding genioplasty is effective at improving both dentofacial deformities and impaired respiratory function.

## Background

Temporomandibular joint (TMJ) ankylosis, often caused by trauma or infection, is a joint disorder characterized by bony or fibrous adhesion of the anatomic joint components, with ensuing loss of function [1]. When this disease occurs in a growing child, it can lead to micrognathia [2]. This type of facial deformity causes narrowing of the upper airway space, and the resulting mechanical obstruction of respiration may provoke obstructive sleep apnea (OSA) [3].

Mandibular distraction osteogenesis (DO), which has now been proposed as an alternative to bilateral sagittal split osteotomy (BSSO), is an effective method for expanding the pharyngeal airways of pediatric [4] and adult patients [5, 6]. It has been shown that mandibular DO results in significant changes in pharyngeal airway volume [6] and the apnea-hypopnea index (AHI) [5, 7] because more than half of upper pharyngeal airway obstructions occur at the base of the tongue [8]. Mandibular DO, which induces gradual movement of skeletal bone and slow stretching of the soft tissue, is considered to reduce the risk of relapse. However, postoperative relapse, which manifests as backward and downward rotation of the mandible, occurs when advancements of greater magnitudes are performed.

It remains unclear how to achieve a stable result for a patient who requires a large mandibular advancement [9, 10]. In recent times, skeletal anchorage, for example, by means of miniscrews, has been widely used to enhance anchorage in craniofacial surgery applications [11–13] because it has been reported that use of such devices after mandibular setback surgery helps to prevent an increase in lower facial height [13]. However, there have been few reports of mandibular DO using miniscrews and miniplates, which are efficient tools for preventing backward and downward rotation of the mandible.

In this case report, we describe successful treatment outcomes and stability of mandibular DO with sliding genioplasty, using skeletal anchorages, in a patient with micrognathia, TMJ ankylosis, and OSA.

## Case presentation

### Diagnosis and etiology

**Fig. 1:**
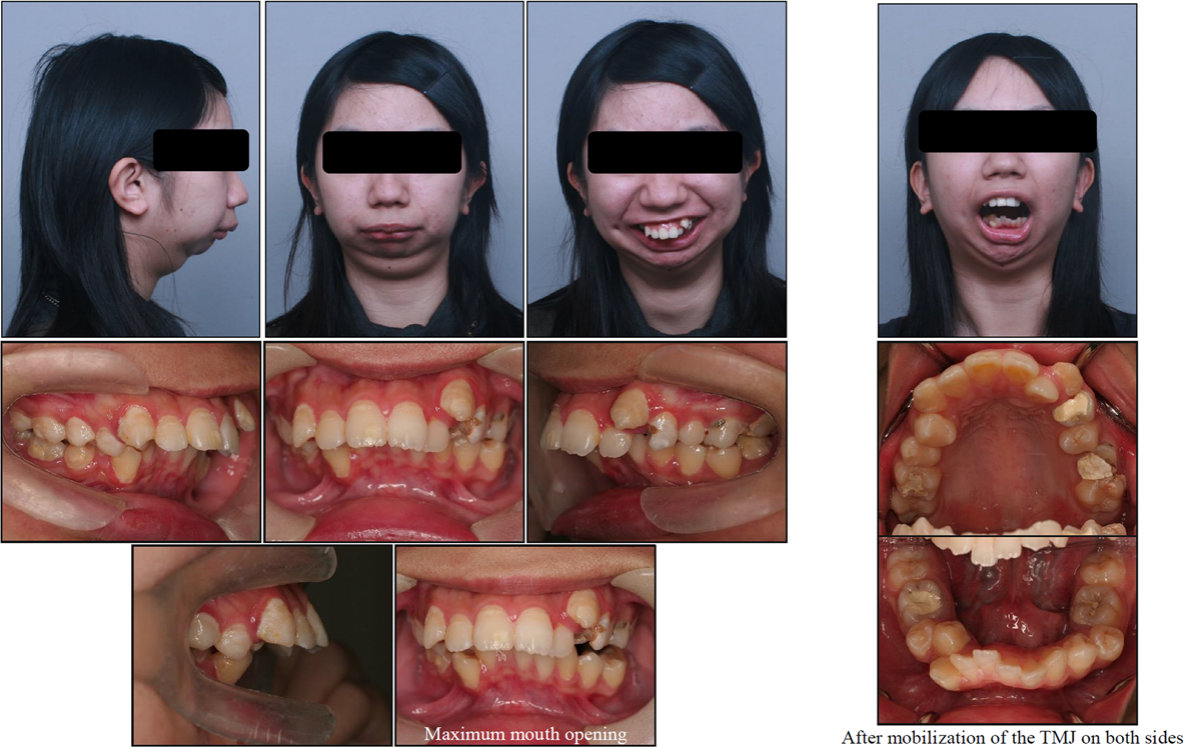
Facial and intraoral photographs at pre-treatment and after mobilization of TMJ ankylosis

A female patient aged 16 years and 11 months presented with severely restricted mouth opening, difficulty in chewing, snoring, and mandibular retrognathism. She was barely able to open her mouth (maximum mouth opening approximately 5.0 mm) (Fig. 1). She showed poor sucking reflex from just after birth and mandibular retrognathia was observed by her mother at 6 months of age, although she had no obvious traumatic history. She was also diagnosed with congenital dislocation of the hip joint at 8 months of age. Anamnesis suggested that her trismus was due to TMJ ankylosis. She had undergone bilateral mobilization of the TMJ at the age of 2 years; however, she had experienced a relapse of impaired mouth opening. She was underweight, with a body mass index of 16.0. Her facial profile was convex with severe micrognathia, her maxillary and mandibular incisors extruded significantly, and she had a gummy smile (Fig. 1).

**Fig. 2:**
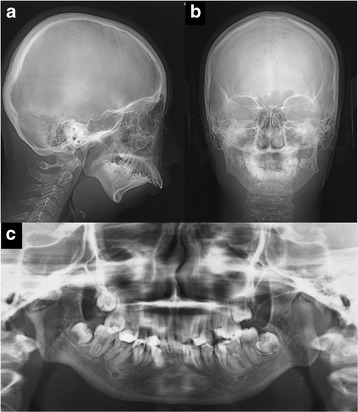
Pre-treatment radiographs. **a** Lateral cephalogram, **b** Posteroanterior cephalogram, and **c** Panoramic radiograph

Cephalometric analysis indicated a severe skeletal Class II malocclusion, with a reduced SNB angle of 62.5° and an increased ANB angle of 18.0° (Fig. 2, Table 1). The mandibular plane angle was extremely steep (mandibular plane-Frankfort horizontal plane = 45.0°). Computed tomography images showed hypoplasia and ankylosis on both sides of the condyle. Her overjet and overbite were 15.0 and 7.0 mm, respectively, and the molar relationship on both sides was Angle Class II (Fig. 1). The arch length discrepancies were −10.5 mm in the maxilla and −12.0 mm in the mandible.

**Table 1:** Cephalometric summary

	Pre-treatment	6 months after mandibular DO	Post-treatment	Japanese female adults norm	
Measurements	Age 16 y 11 mo.	Age 20 y 1 mo.	Age 22 y 1 mo.	Mean	SD
Angular (°)					
ANB	18.0	13.0	13.0	3.3	2.7
SNA	80.5	80.5	80.5	80.8	3.6
SNB	62.5	67.5	67.5	77.9	3.6
Facial angle	64.0	68.0	69.5	84.8	3.0
Gonial angle	144.0	140.0	141.0	122.1	5.3
Mand pl-FH pl	45.0	49.0	50.0	30.5	3.6
U1-FH pl	106.5	91.5	92.5	112.3	8.3
L1-mand pl	111.0	116.0	107.0	93.4	6.8
Interincisal angle	97.5	103.5	110.5	123.6	10.6
Linear (mm)					
S-N	76.0	76.0	76.0	67.9	3.7
N-Me	115.5	118.5	122.0	125.8	5.0
Me/palatal pl	53.0	59.5	63.5	68.6	3.7
Go-Me	47.0	57.5	62.0	71.4	4.1
U1 to APO	22.0	12.0	11.5	6.2	1.5
L1 to APO	24.0	12.5	9.0	3.0	1.5
Overjet	15.0	0.0	2.5	3.1	1.1
Overbite	7.0	0.0	1.5	3.3	1.9
Soft tissue (mm)					
Upper lip to E-line	11.5	2.5	0.0	−2.5	1.9
Lower lip to E-line	8.5	3.5	−1.5	0.9	1.9
Upper airway length	70.5		62.0		
Hyoid bone position	33.8	_	22.2	_	_

*Mand* mandibular, *pl* plane, *FH* Frankfort horizontal

Upper airway length: the vertical airway length

Hyoid bone position: hyoid-to-mandibular plane distance

The patient underwent standard polysomnography, which revealed a high AHI, frequent arousals, and depression of lowest oxygen saturation (SaO_2_) during sleep (Table 2).

**Table 2:** Standard polysomnography evaluation

Variables	Pre-treatment	Post-treatment
Apnea hypopnea index (N/h)	37.1	8.7
Lowest SaO_2_ (%)	71.0	87.0
Arousal index (N/h)	41.2	25.0
Sleep efficiency (%)	94.0	96.3

*SaO*_*2*_ oxygen saturation

### Treatment objectives

The patient was diagnosed with an Angle Class II malocclusion with severe skeletal Class II, hypoplasia and ankylosis of the TMJ on both sides, and severe OSA. The treatment objectives were as follows: (1) to improve the TMJ ankylosis and impaired mouth opening by mobilizing the TMJ on both sides; (2) to correct the mandibular retrusion and improve the retrognathic appearance of the facial profile by mandibular DO, using miniscrews and miniplates to prevent extrusion of the upper and lower teeth; (3) to bring the tip of the mandible forward by sliding genioplasty; (4) to resolve the severe crowding of the upper and lower anterior segment by extraction of all first premolars and a large damaged upper first molar on the left side; (5) to establish good functional occlusion by achieving an Angle Class I occlusion, and ideal overjet and overbite; (6) to extract the impacted upper third molar on the right side and the impacted lower third molars on both sides; and (7) to expand the upper airway space three-dimensionally by mandibular DO with sliding genioplasty.

### Treatment procedure

Before orthodontic treatment, the patient underwent interpositional arthroplasty. Briefly, an interposition of fascia temporalis and surrounding fat tissue was inserted into the defect after bilateral condylectomy. Bilateral condylar bone was resected at a width of 8 mm at the level of incisura of the mandible. Intra- and postoperative incisal opening of more than 30 mm, and no obvious occlusal changes, were confirmed (Fig. 1). Nine months later, all first premolars, the maxillary first molar on the left side, maxillary third molar on the right side, and mandibular third molars were extracted under general anesthesia. A miniscrew (2.0 mm in diameter, 8.0 mm in length; Dual-Top, Jeil Medical Corporation, Seoul, Korea) was implanted into the maxillary alveolar bone between the premolars and first molars on the right side to reinforce anchorage. A preadjusted edgewise appliance (0.018 × 0.025-in. slot) was applied at the maxillary and mandibular arches. After leveling and alignment with nickel-titanium arch wires, canine retraction was initiated using stainless steel round wire. The maxillary incisors were retracted using sliding mechanics. The preoperative orthodontic treatment period was 1 year and 7 months.

**Fig. 3:**
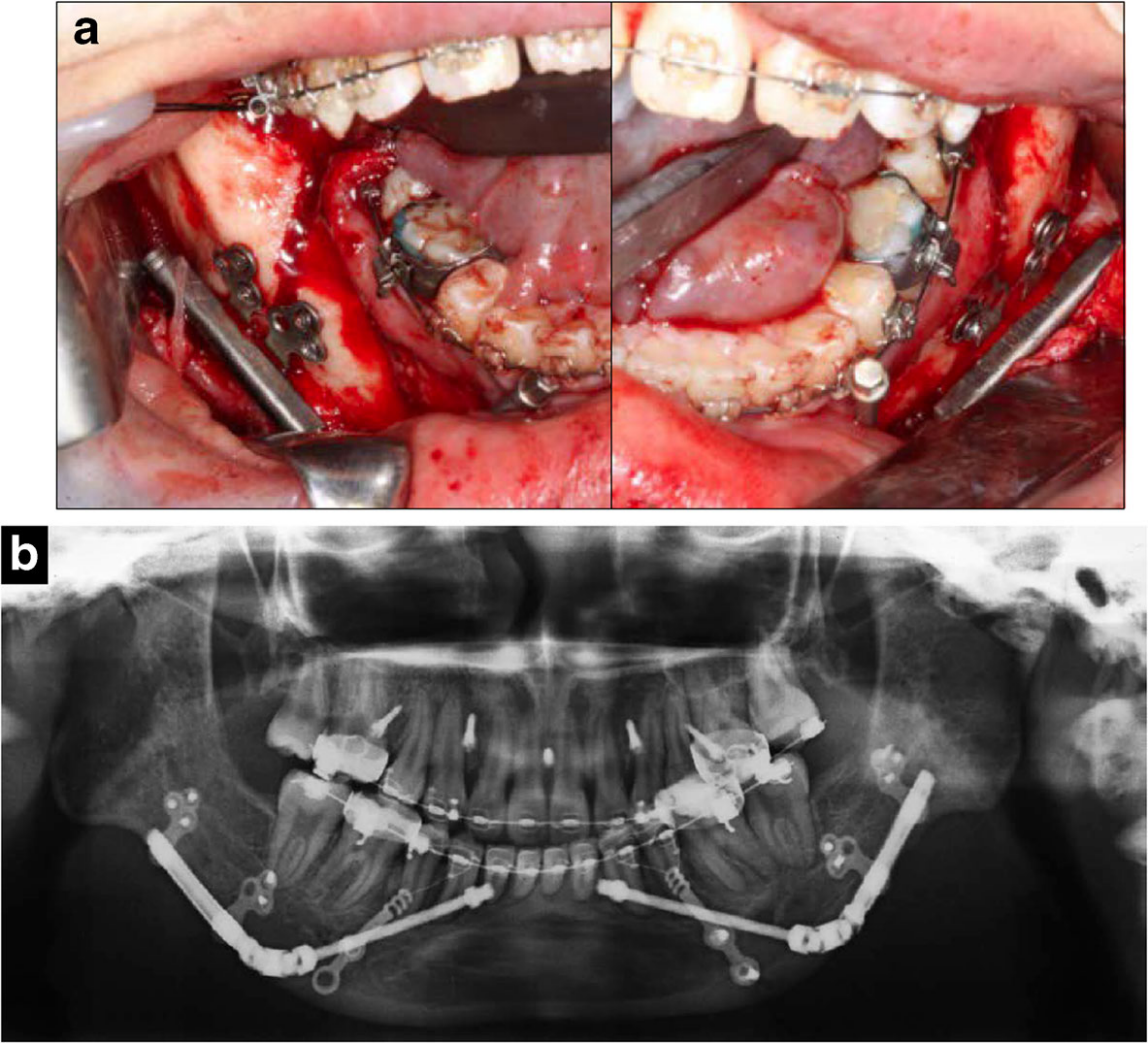
**a** Distraction devices applied to the posterior region of the mandible body bilaterally. **b** Panoramic radiograph after mandibular distraction osteogenesis

**Fig. 4:**
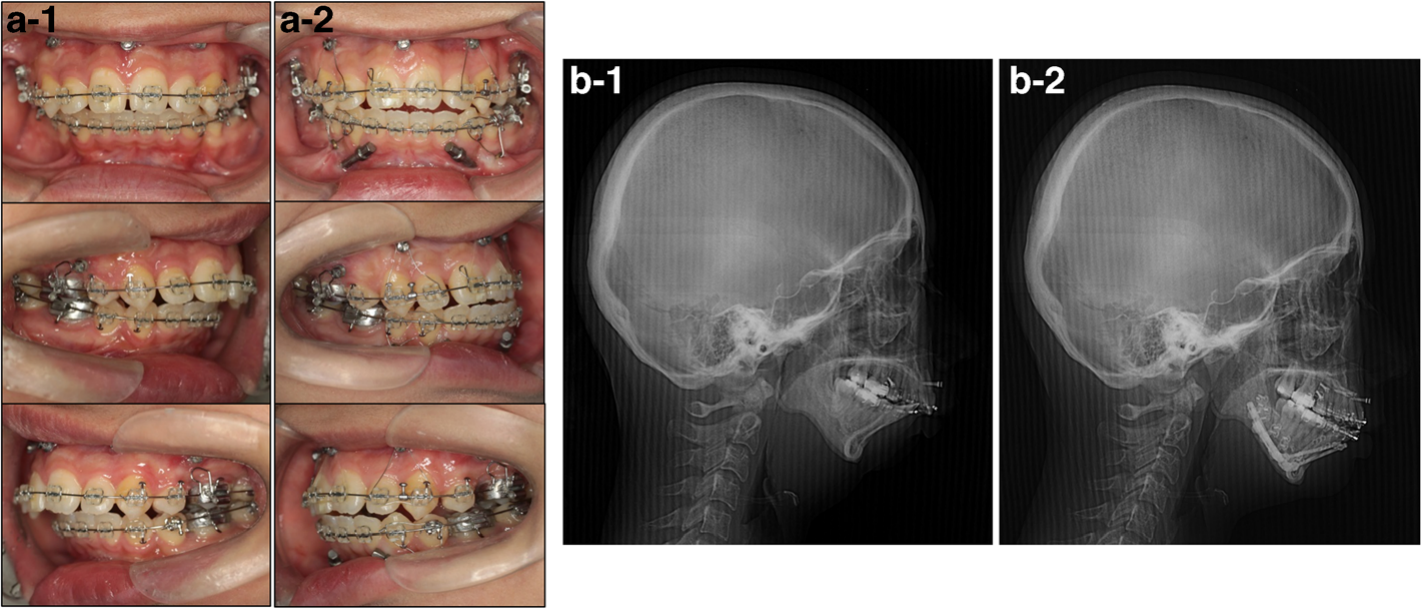
**a** Intraoral photographs obtained immediately before (a-1) and after (a-2) mandibular distraction osteogenesis. **b** Lateral cephalograms obtained immediately before (b-1) and after (b-2) mandibular distraction osteogenesis

After completion of the preoperative orthodontic treatment, miniscrews (2.0 mm in diameter, 8.0 mm in length; Dual-Top) were implanted between the central incisors, and between the lateral incisors and canines on both sides of the maxilla. After extraction of the third molars at 1 year and 9 months, mandibular DO was performed using distraction devices (Zürich Pediatric Ramus Distractor, cloverleaf design, KLS Martin, Tuttlingen, Germany) applied to the posterior region of the body of the mandible, bilaterally (Fig. 3). First, a buccal vertical corticotomy was made using a Lindemann bur in the third molar region without exposure of the inferior alveolar nerve. After removal of the adapted distractor, the osteotomy was completed by fracture of the lingual cortex. Finally, Smith forceps were inserted into the buccal osteotomy and mobilization was then confirmed. Furthermore, miniplates (25.0 mm in length; Dentsply, Tokyo, Japan) were implanted between the canines and premolars in the mandibular region of the anterior alveolar bone because the interradicular spaces of these areas on both sides appeared to be narrow on a panoramic radiograph. The distraction device was activated twice a day at a rate of 1.0 mm per day for 17 days, until edge-to-edge occlusion was achieved, including overcorrection (Fig. 4). Neurosensory disturbance of the inferior alveolar nerve did not occur after advancement of the mandible. Intermaxillary elastics were used with the miniscrews and miniplates to reduce the downward and backward rotation of the mandible during mandibular elongation. The total amount of mandibular advancement was 15.0 mm on the left side and 11.5 mm on the right.

**Fig. 5:**
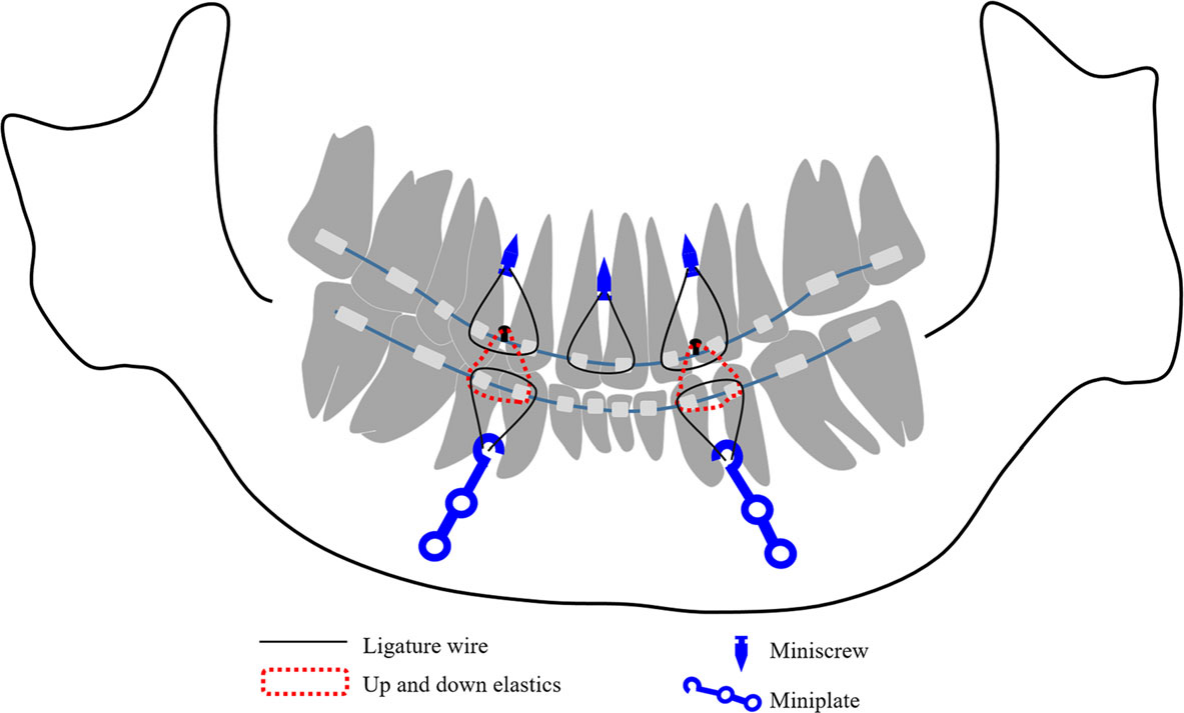
Schematic illustration of the use of up-and-down elastics with miniscrews and miniplates for 7 months

After mandibular DO, the miniscrews and miniplates linked the canine and first premolar with a 0.25-mm ligature wire to prevent extrusion of the upper and lower teeth, while using up-and-down elastics for 7 months (Fig. 5). Sliding genioplasty was performed to bring the tip of the mandible forward when the distraction devices were removed. The mandibular distraction device was maintained in position post-distraction for 7 months. The total duration of active orthodontic treatment was 4 years and 7 months, and lingual bonded retainers were placed in the maxillary and mandibular arch immediately after removal of the edgewise appliance.

### Treatment outcomes

**Fig. 6:**
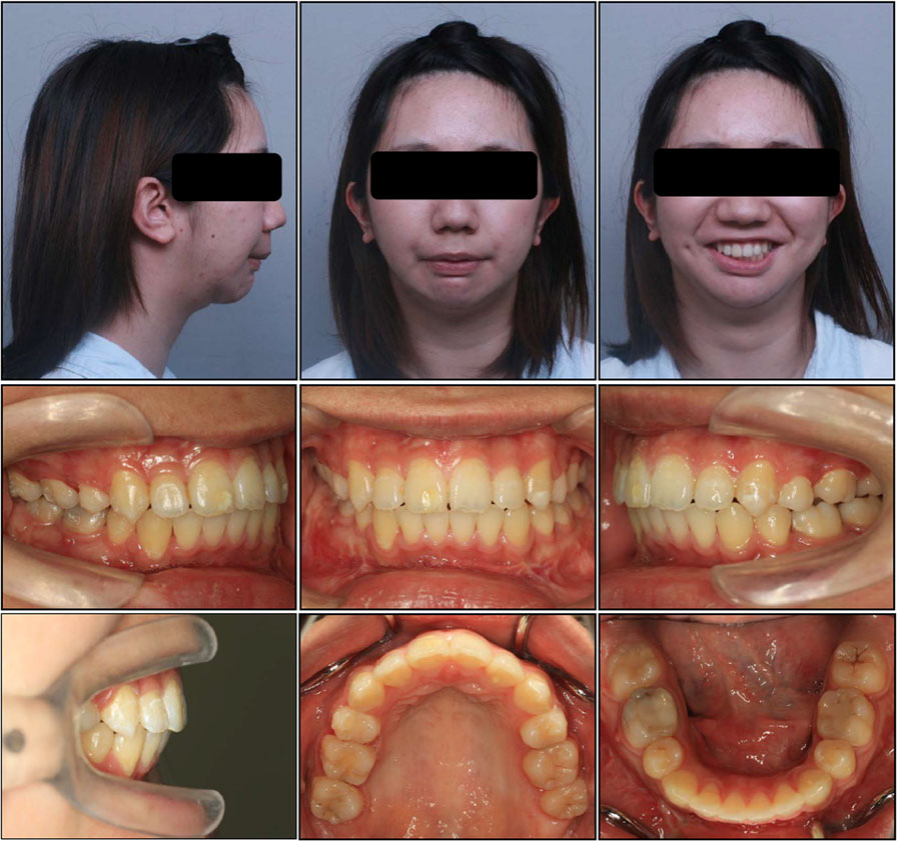
Post-treatment facial and intraoral photographs

**Fig. 7:**
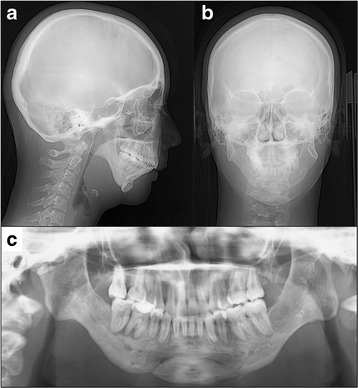
Post-treatment radiographs. **a** Lateral cephalogram, **b** Posteroanterior cephalogram, and **c** Panoramic radiograph

Post-treatment facial photographs showed significant improvement of the convex profile and gummy smile (Fig. 6). The maxillary incisor was lingually inclined by 14.0° and intruded by 2.0 mm, and the mandibular incisor was lingually inclined by 4.0° and intruded by 2.0 mm (Fig. 7, Table 1). The large overjet and deep overbite were corrected to 2.5 and 1.5 mm, respectively, and good intercuspation with an Angle Class I molar relationship was achieved.

**Fig. 8:**
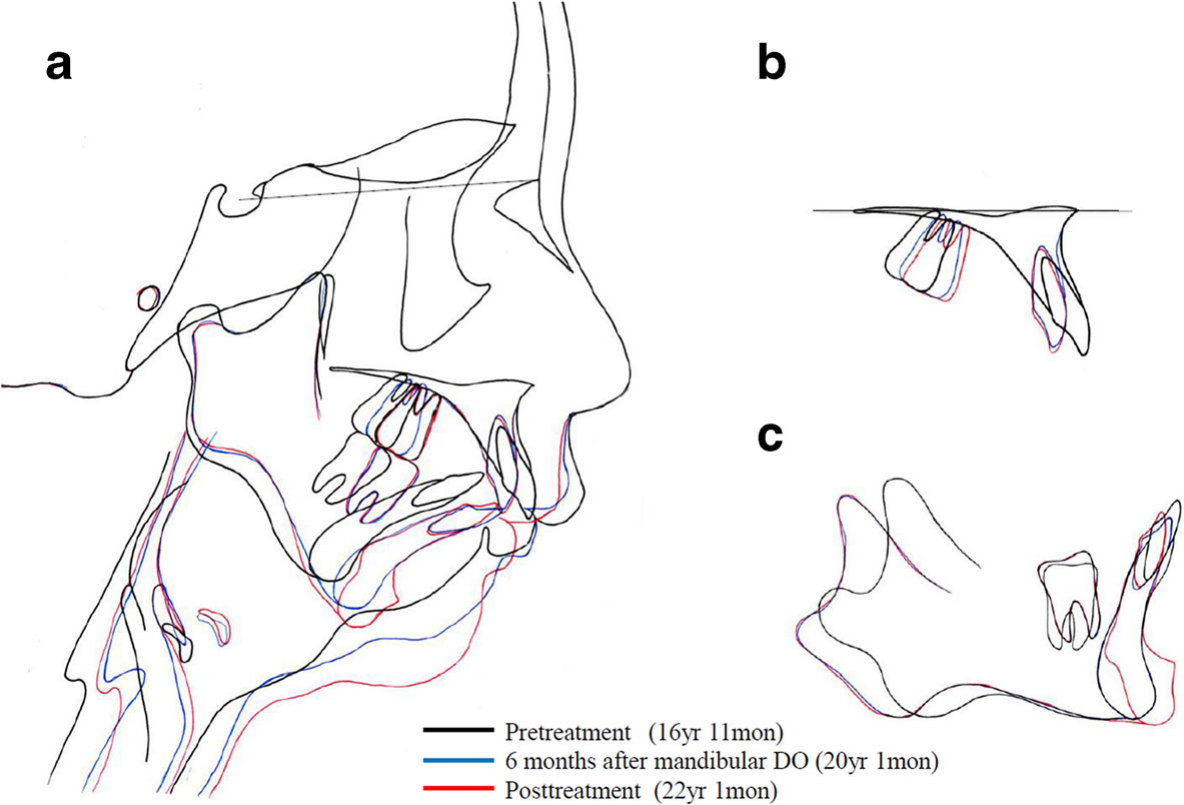
Superimposition of cephalometric tracings at pre-treatment, 6 months after mandibular DO, and post-treatment. **a** Sella-nasion plane at the sella, **b** Anterior palatal contour, and **c** Regional mandibular anatomy

Cephalometric evaluation at 6 months after mandibular DO showed mandibular advancement of 11.5 mm at the menton (Fig. 8). At 6 months after mandibular DO, the total relapse of the pogonion in the anteroposterior direction was 4.7% of the mandibular advancement. Accordingly, the SNB angle and facial angle increased by 5.0° and 4.0°, respectively. Furthermore, the chin was moved superiorly by approximately 4.5 mm with sliding genioplasty. The total amount of mandibular advancement was 16.0 mm at the menton (Fig. 7).

**Fig. 9:**
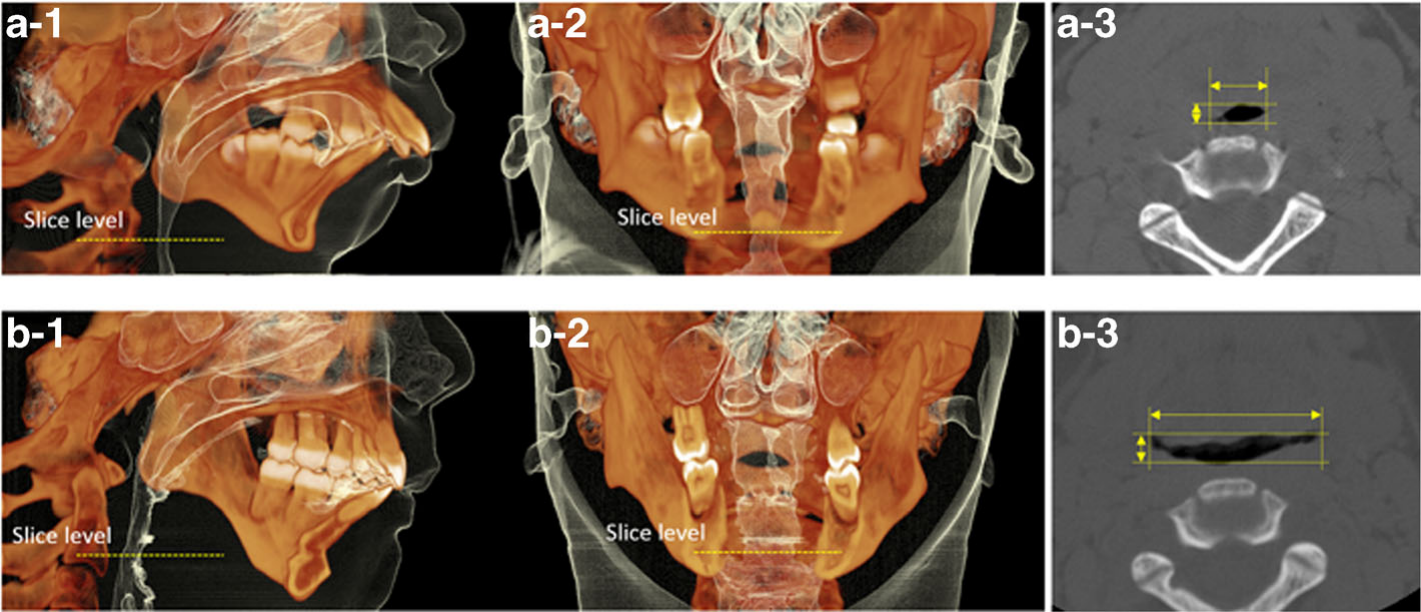
Volume rendering computed tomography images of the upper airway. Lateral view (a-1), frontal view (a-2), and transverse and anteroposterior airway diameters in the retroglossal region (a-3) at pre-treatment; lateral view (b-1), and frontal view (b-2), transverse and anteroposterior airway diameters in the retroglossal region (b-3) at post-treatment

To examine the three-dimensional morphologic changes in the upper airway, we analyzed the volume data for the upper airway, using volume-rendering software (CephaloMetrics AtoZ, v. 16; Yasunaga, Tokyo, Japan). Although the anteroposterior diameter of the upper airway had increased only slightly, the transverse diameter significantly increased in the retroglossal area (Fig. 9). Additionally, the airway length and hyoid bone position decreased from 70.5 to 62.0 mm and from 33.8 to 22.2 mm, respectively (Table 1).

Polysomnography showed that the AHI had decreased from 37.1 to 8.7 N/h, the lowest SaO_2_ during sleep increased from 71.0 to 87.0%, and the arousal index decreased from 41.2 to 25.0 N/h (Table 2). The severity of OSA and snoring significantly improved after orthognathic surgery.

**Fig. 10:**
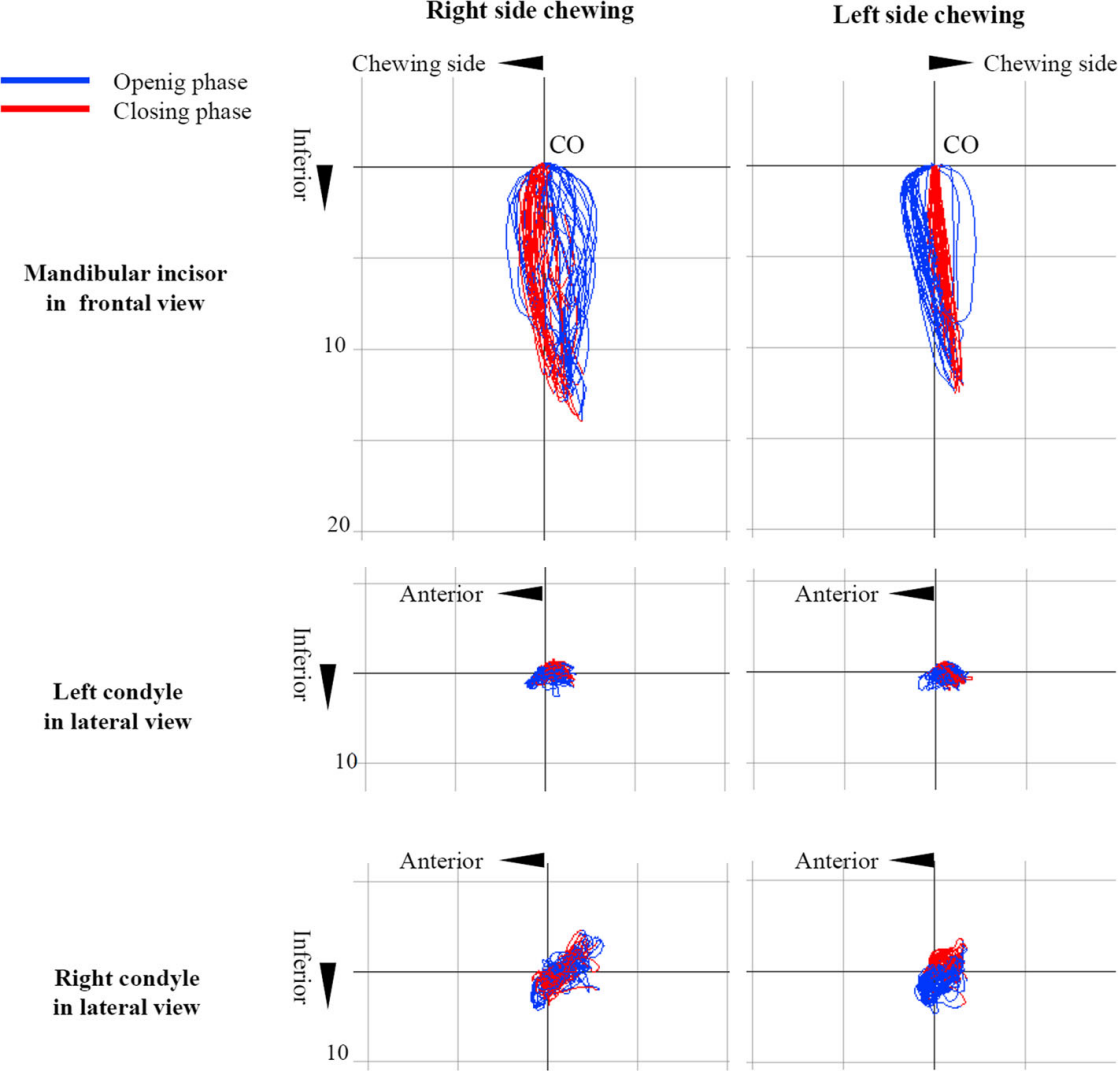
Jaw movement of the lower central incisor and condyle during unilateral gum chewing on both sides, post-treatment. CO indicates the position at each measuring point in the maximum intercuspation position

Masticatory jaw movement during unilateral gum chewing was recorded using an optoelectric jaw-tracking system with 6 degrees of freedom (Gnatho-Hexagraph II; GC International, Tokyo, Japan) after orthognathic surgery. There were significant improvements in the severely limited mouth opening and impaired masticatory function (Fig. 10, Table 3). There was no obvious bone resorption or pain in the temporomandibular region, limited mouth opening (maximum mouth opening: 33.0 mm), myofascial pain or headache, downward rotation of the mandible, or lateral shift of mandibular position evident at 5 years and 6 months after mandibular DO.

**Table 3:** Jaw movement variables during unilateral gum chewing on both sides post treatment

Jaw movement variables	Right side	Left side
	Mean SD	Mean SD
Cycle duration (ms)	1014 ± 280	751 ± 212
Maximum closing velocity (mm/sec)	53.4 ± 11	67.9 ± 14
Maximum gap (mm)	11.3 ± 2.9	9.7 ± 2
Cycle width (mm)	2.9 ± 0.7	2.3 ± 0.5

## Discussion

This case report documents successful treatment outcomes and stability of mandibular DO with sliding genioplasty, using skeletal anchorages, in a patient with micrognathia, TMJ ankylosis, and OSA. The retrognathic appearance of the patient’s facial profile and severe OSA required orthognathic surgery. Several procedures were considered for achieving an appropriate facial profile and acceptable occlusion. On the basis of the treatment objectives, the following alternatives were considered: mandibular DO with sliding genioplasty, BSSO and immediate movement of the mandible forwards with sliding genioplasty, and maxillomandibular advancement surgery of the maxillomandibular complex with increasing the length of the mandibular ramus, altering the occlusal plane. The amount of mandibular advancement required exceeded 10 mm. The second option was rejected because, compared with BSSO, mandibular DO significantly reduces the incidence of neurosensory disturbance of the inferior alveolar nerve and the risk of relapse is lower in the case of large mandibular advancement (> 7 mm) [9, 10]. It has been suggested that the smallest preoperative cross-sectional area of the upper airway should be considered for preoperative selection of the maxilla or mandible, or both, for advancement because there is a significant relationship between the narrowest cross-section of the upper airway and the probability of OSA [14]. In this case, the narrow area of the upper airway was the retroglossal section rather than the retropalatal area, owing to the small mandible and the normal maxilla (SNA angle within the normal range). Therefore, the third treatment option was rejected because a small retroglossal area can be entirely corrected by mandibular DO with sliding genioplasty, as previously reported [4–7].

The primary cause of postoperative instability after mandibular advancement surgery is the tension produced by the elongated and stretched suprahyoid muscles, and associated connective tissues [15]. It has been reported that counter-clockwise rotation can occur during advancement in high-angle cases and that the duration of relapse tends to be longer in cases with larger advancements [16, 17]. To reduce these side effects, up-and-down elastics or short Class II elastics should be applied to the anterior teeth immediately during and after surgical advancement of the mandible. However, if vertical or short Class II elastics are applied to the anterior teeth for an extended period, there is a tendency for extrusion of the teeth. These dental changes cause downward and backward rotation of the mandible, and it is then impossible to prevent postoperative skeletal relapse, which worsens the maxillomandibular relationship.

Recently, miniscrews have been used to provide absolute anchorage in various types of procedures involving tooth movement [18]. In the present case, miniscrews and miniplates were placed in the maxillomandibular region of the anterior alveolar bone, and the mandible was then pulled in an anterosuperior direction, using intermaxillary elastics between the miniscrews and miniplates, over a period of 7 months after mandibular DO. Consequently, the anterior and posterior teeth were not extruded, and the change in the pogonion at 24 months post-treatment (4 years and 6 months after mandibular DO surgery) was 4.7% in the anteroposterior direction, which was less than previously reported [19]. The amount of downward rotation of the mandible during advancement was negligible; the mandibular plane angle increased by only 4.0°. The skeletal stability was due to the use of intermaxillary elastics for more than 6 months and the minimal tooth movements resulting from use of skeletal anchorage after mandibular DO.

Despite abnormal maxillomandibular relationships, the soft tissues and bones of the craniofacial complex are essentially in a balanced, homeostatic condition [20]. After lengthening, the patent mandible begins to establish a new balanced, homeostatic condition. Consequently, a sufficiently long period of postoperative traction of the mandible may be required to allow the masticatory system to adapt to the new perimandibular soft tissue environment. Therefore, to achieve dental stability in terms of the vertical position of the maxilla and mandible for a patient with a high angle who requires large advancement of the mandible, intermaxillary elastics with miniscrews and/or miniplates may be used to continue to prevent downward and backward rotation of the mandible for a period exceeding 6 months.

The commonly accepted criteria for surgical success in patients with OSA are a 50% reduction in the AHI index and less than 20 events per hour, with a near normal peripheral SaO_2_ [21–23]. In the present case, overnight respiratory function before and after surgery showed that the AHI decreased from 37.1 to 8.7 N/h (75% reduction), the lowest SaO_2_ during sleep increased from 71.0 to 87.0%, and the arousal index decreased from 41.2 to 25.0 N/h. This indicated significant improvement of the severe OSA, although the AHI was not reduced to within the normal range (AHI < 5).

**Fig. 11:**
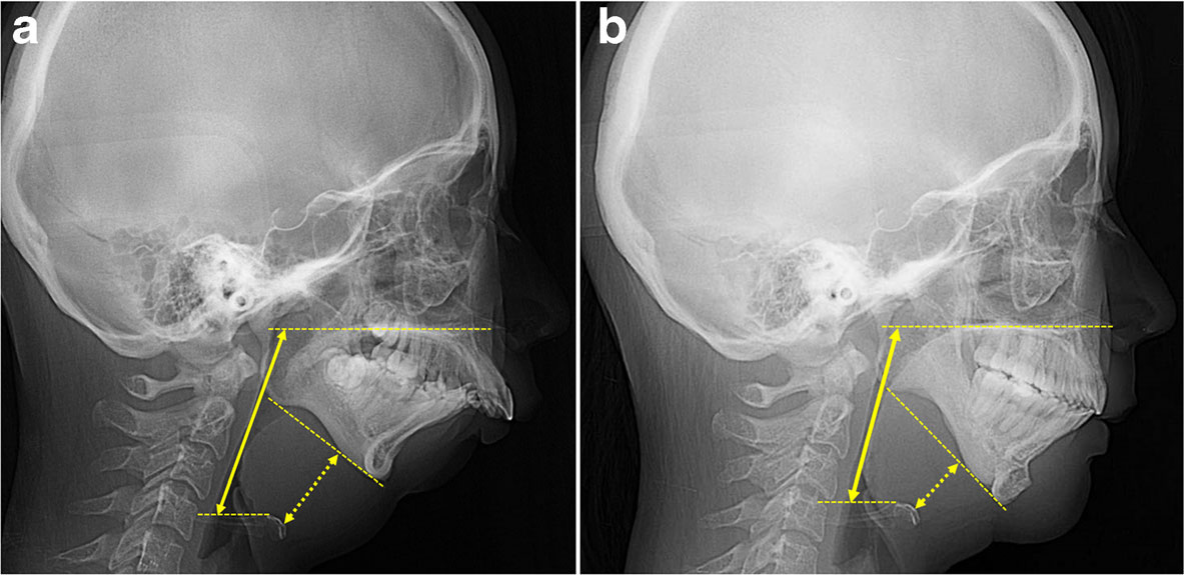
Measurement of airway length and hyoid bone to mandibular plane distance pre-treatment (**a**) and post-treatment (**b**). *Solid line*: airway length between a horizontal plane tangent to the superior aspect of the hyoid bone and a horizontal plane tangent to the posterior palate, parallel to the long axis of the airway. *Dotted line*: hyoid bone to mandibular plane; the most anterosuperior point of the greater cornu of the hyoid bone on the line perpendicular to the mandibular plane

Recently, it has been reported that significant improvement can be achieved in adult patients by surgically managing OSA with mandibular advancement procedures, such as DO [5]. Mandibular DO results in an increase in the retroglossal airway volume by increasing the space within the mandible and exerts forward traction over the tongue musculature. According to the three-dimensional retroglossal airway image of this case, the airway was increased more laterally than antero-posteriorly, which is similar to previous reports stating that lateral dimensions are more enhanced than anteroposterior dimensions in the retroglossal region [5, 14] (Fig. 9). Additionally, both the airway length and hyoid bone position reduced (by 12.1 and 34.3%, respectively) after orthognathic surgery (Table 4, Fig. 11). It is thought that mandibular DO with sliding genioplasty pulled the geniohyoid and genioglossus muscles upward and forward, causing an upward and forward shift of the tongue base and the hyoid bone. Resistance to air flow is related to airway diameter and length (Pouseuille’s Law). In this case, the three-dimensionally expanded upper airway space and reduced airway length after surgery appears to have resulted in decreased airway resistance.

**Table 4:** Measurement of pharyngeal airway

Variables	Pretreatment	Posttreatment	Change (%)
Cross-sectional area of the narrowest upper airway (mm^2^)	15.9	38.7	143.3
Anteroposterior diameter (mm)	3.1	3.7	19.4
Transverse diameter (mm)	11.0	22.4	103.6
Upper airway length(mm)	70.5	62.0	−12.1
Hyoid bone position(mm)	33.8	22.2	−34.3

Upper airway length: hyoid-to-palatal plane distance

Hyoid bone position: hyoid-to-mandibular plane distance

Change (%): (postoperative value - preoperative value) / preoperative value × 100

The overnight respiratory function results and morphologic changes in the pharyngeal airway suggest that mandibular DO with sliding genioplasty can effectively improve not only micrognathia, but also severe OSA. However, the lateral cephalograms and cone-beam computed tomography data were obtained for a patient sitting upright, with the head, tongue, and peripharyngeal soft tissues in a neutral position. Reproducible head and tongue positions need to be defined and should be included in such evaluations. The postoperative oropharyngeal airway and AHI in the present case were not improved to within the normal range, and there may have been a risk of mouth opening reduction and TMJ pain. Continued follow-up observations of respiratory function and TMJ condition are therefore required.

## Conclusions

Mandibular DO using skeletal anchorage with intermaxillary elastics is a useful option for preventing extrusion of the upper and lower anterior teeth, thus helping to prevent downward and backward rotation of the mandible. In addition, mandibular DO combined with sliding genioplasty may be an effective method for improving not only dentofacial deformities, but also impaired respiratory function by changing the shape and length of the upper airway.

## Abbreviations

AHI: Apnea-hypopnea index
BSSO: Bilateral sagittal split osteotomy
DO: Distraction osteogenesis
OSA: Obstructive sleep apnea
SaO_2_: Oxygen saturation
TMJ: Temporomandibular joint
